# Pathogenesis and Treatment of Pruritus Associated with Chronic Kidney Disease and Cholestasis

**DOI:** 10.3390/ijms24021559

**Published:** 2023-01-13

**Authors:** Jin-Cheol Kim, Won-Sik Shim, In-Suk Kwak, Dong-Hun Lee, Jin-Seo Park, So-Yeon Lee, Seok-Young Kang, Bo-Young Chung, Chun-Wook Park, Hye-One Kim

**Affiliations:** 1 Department of Dermatology, Kangnam Sacred Heart Hospital, Hallym University, Seoul 07440, Republic of Korea; 2 College of Pharmacy, Gachon University, Incheon 21936, Republic of Korea; 3 Department of Anesthesiology and Pain Medicine, Burn Center, Hangang Sacred Heart Hospital, Hallym University, Seoul 07247, Republic of Korea; 4 Department of Dermatology, Seoul National University Hospital, Seoul National University College of Medicine, Seoul 03080, Republic of Korea

**Keywords:** pruritus, systemic disease, chronic kidney disease, cholestasis

## Abstract

Itching is an unpleasant sensation that provokes the desire to scratch. In general, itching is caused by dermatologic diseases, but it can also be caused by systemic diseases. Since itching hampers patients’ quality of life, it is important to understand the appropriate treatment and pathophysiology of pruritus caused by systemic diseases to improve the quality of life. Mechanisms are being studied through animal or human studies, and various treatments are being tested through clinical trials. We report current trends of two major systemic diseases: chronic kidney disease and cholestatic liver disease. This review summarizes the causes and pathophysiology of systemic diseases with pruritus and appropriate treatments. This article will contribute to patients’ quality of life. Further research will help understand the mechanisms and develop new strategies in the future.

## 1. Introduction

Itching is an unpleasant sensation that provokes the desire to scratch. Itching is one of the factors that hamper patients’ quality of life due to disturbances in daily activities and sleep. Skin lesions are normally worsened by scratching, also known as the “itch–scratch cycle”. In general, itching is thought to be caused by dermatologic diseases such as atopic disease, contact dermatitis, and xerotic eczema; however, it can also be caused by systemic diseases such as chronic kidney disease, cholestatic liver disease, and thyroid diseases. Pruritus caused by systemic diseases improves as the systemic diseases are treated, but chronic manifestations may still remain. Therefore, it is important to understand the appropriate treatment and pathophysiology of pruritus caused by systemic diseases to improve the patients’ quality of life.

Chronic kidney disease (CKD)-associated pruritus is a common complication experienced by CKD patients on blood or peritoneal dialysis. Cholestasis is another condition frequently accompanied by itching, as well as jaundice and dark urine due to increased bilirubin. The incidence of cholestatic pruritus varies depending on the underlying diseases.

This review will focus on pruritus associated with CKD and cholestatic liver disease. This review reports the causes and pathophysiology of systemic diseases with pruritus and appropriate treatments with current trends. This review intends to present the latest findings of recent studies.

## 2. Clinical Feature of CKD-Associated Pruritus

### 2.1. CKD-Associated Pruritus and Patients Characteristics

CKD is defined as a spectrum of pathophysiologic processes associated with kidney damage or decreased glomerular filtration rate for three or more months, irrespective of the causes. Pruritus in CKD patients, also known as uremic itch, is a common symptom affecting 37–64% of patients worldwide [1,2,3]. The prevalence of uremic pruritus in adult hemodialysis patients has varied over time, and recent reports suggest a decreasing trend with the development of an effective dialysis method: high-flux hemodialysis [4]. As a result of the Dialysis Outcomes and Practice Patterns Study (DOPPS) trial, the percentage of patients with extreme pruritus dropped from 28 to 18 percent between 1996–2001 and 2012–2015 [3,5]. Its prevalence is not well studied in adults or pediatric patients on peritoneal dialysis. A small number of studies suggested that the prevalence is lower in children compared to adults. Two studies showed conflicting results in the prevalence of pruritus in hemodialysis and peritoneal dialysis patients [6,7,8]. In addition to the underlying CKD, various risk factors have turned out to be associated with uremic pruritus in observational studies (Table 1). Supportive therapies have been suggested to treat such factors.

### 2.2. Clinical Manifestations of CKD-Associated Pruritus

**Figure 1:**
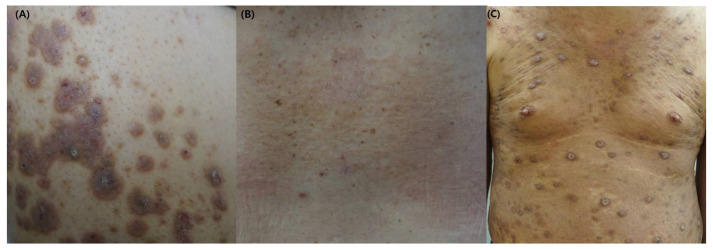
The clinical images of patients with CKD-associated pruritus (**A**) Multiple excoriated, purple-colored nodules or plaques on the back. (**B**) Scaly, erythematous patches on the back. (**C**) Multiple excoriated, dome-shaped nodules on the trunk.

CKD-associated pruritus most commonly affects the posterior trunk but may also involve the arms, head, and anterior trunk [5,21]. The symptoms tend to be more severe at night, resulting in sleep disruption [5,9,22], fatigue, and depression [3,5]. The pruritus can increase in hot temperature, stress, or during hemodialysis sessions [4,15]. Patients with pruritus show abnormal laboratory results, including elevated serum blood urea nitrogen (BUN), phosphate, parathyroid hormone, and calcium level [9]. Hemodialysis patients do not have primary but secondary scratch lesions due to the characteristics of systemic disease (Figure 1) [2,23,24]. Physical findings are limited unless repeated scratching develops into lichen simplex or prurigo nodularis [25]. Since pruritus is common in dialysis patients, it can be diagnosed by excluding the pruritus caused by other diseases. However, it should be noted that some patients can have pruritus due to the exacerbation of accompanying dermatological disorders, drug allergy, lymphoma, or cholestasis.

### 2.3. The Quality of Life Affected by CKD-Associated Pruritus

One prospective cohort study, the GEHIS (German Epidemiological Hemodialysis Itch Study), started in 2013 with 860 hemodialyses (HD) patients [26]. The study investigated the prevalence, intensity, and characteristics of itch with quality of life by evaluating it based on a short-form health survey (SF-12) and a hospital anxiety and depression scale (HADS). It also examined the skin states, including dermatologic disorders and secondary lesions classified by the International Forum for the Study of Itch (IFSI) [26]. 

IFSI classification divided pruritus into three classes according to the causes and skin lesions. IFSI clinical I means pruritus caused by primary dermatologic diseases. IFSI clinical II means pruritus with normal skin, and IFSI clinical III means pruritus with secondary skin lesions. There was no significant difference in prognosis of patients with and without an itch. However, the severity of the itch has proved to be an important marker for poor prognosis [9]. Patients with a chronic itch who had secondary scratch lesions and chronic prurigo (IFSI clinical III) showed significantly increased mortality after controlling for age and sex in a multivariate model. In addition, patients with secondary scratch lesions showed a higher severity of itch and lower quality of life.

## 3. Pathogenesis of CKD-Associated Pruritus

Although the pathogenesis of CKD-associated pruritus has not been fully elucidated, several mechanisms have been proposed.

### 3.1. Dysregulation of the Endogenous Opioid System

**Figure 2:**
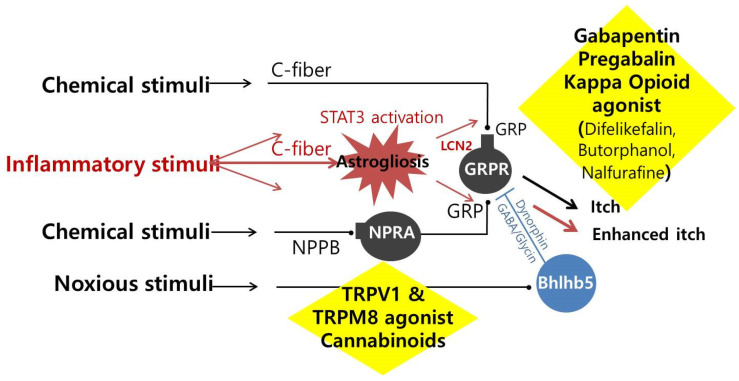
The mechanism of treatment for patients with uremic pruritus STAT3; Signal transducer and activator of transcription 3, GRP; Gastrin-releasing peptide, GRPR; Gastrin-releasing peptide receptor, LCN2; Lipocalin-2, NPRA; Natriuretic peptide receptor-A, NPPB; Natiurtetic polypeptide b, GABA; Gamma-aminobutyric acid, Bhlhb5; Basic helix-loop-helix B 5, TRPV1; Transient receptor potential vanilloid type 1, TRPM8; Transient receptor potential melastatin 8.

The endogenous opioid system in the central nervous system is mainly involved in pain and pruritus. Dysregulation of the opioid system is commonly associated with the pathogenesis of CKD-associated pruritus. The opioid system consists of three significant receptors; μ-opioid receptor (MOR, a receptor for β-endorphin), κ-opioid receptor (KOR, a receptor for dynorphin), and δ-opioid receptor (DOR, a receptor for encephalin). Dynorphin, released from Bhlhb5-positive neurons, inhibits KOR on GRPR neurons, and the dysfunction of the inhibitory circuits can lead to neural sensitization, resulting in reduced pruritus (Figure 2). However, the inhibition is antagonized by MOR activation. A significant decrease in KOR expression was shown in the skin of patients with pruritus [27]. In conclusion, when KOR is activated, itching is suppressed, and conversely, when MOR is activated, itching is promoted [28]. 

In the pathogenesis of CKD-associated pruritus, the over-activation of MOR is suggested to play a role. In hemodialysis patients, itch intensity was positively correlated to the serum concentration of β-endorphin and MOR agonist-to-KOR agonist ratio. In addition, a significant negative correlation between the severity of pruritus and the expression of KOR existed [29]. The MOR antagonist (naloxone, naltrexone) was shown to improve CKD-associated pruritus significantly [30]. In addition, the KOR agonist (nalfurafine, difelikefaline) significantly improved the CKD-associated pruritus and the patients’ quality of life [31].

### 3.2. Uremic Neuropathy

Uremic neuropathy is a well-known mechanism of CKD-associated pruritus [32]. Dialysis patients have altered neurophysiological responses. In patients with uremia, peripheral nerve endings are reduced, and some nerve branches extend irregularly to the epidermis, paradoxically increasing the excitability of the nerve [33,34,35]. Moreover, pruritus can induce scratching, neurogenic inflammation, and destruction of epidermal nerve endings, known as the “itch–scratch cycle”. The chronic itch and scratch can cause astrogliosis in the spinal cord, over-excitability of GRPR neurons, and transitioning to a more severe neurogenic itching state. 

The fact that the medicines impacting the neurotransmission, such as tricyclic antidepressants (amitriptyline), selective serotonin reuptake inhibitors (fluoxetine), and gabapentin, result in a significant improvement in CKD-associated pruritus suggests that neuropathy is involved in the pathogenesis of CKD-associated pruritus.

### 3.3. Alteration of Metabolism

The levels of calcium phosphate (CaP) is commonly increased in the skin of patients with CKD-associated pruritus. Although markers of mineral metabolism and dialysis efficiency were previously thought to be related to CKD-associated pruritus, many studies, including Dialysis Outcomes and Practice Patterns Study (DOPPS), show conflicting results. In DOPPS, a multivariate analysis of 6256 patients showed inconsistent results with no significant correlation between CKD-associated pruritus and the concentration of phosphorus, calcium, calcium-phosphorus products, and parathyroid hormone (PTH). 

In mice, intradermal injection of CaP induced pruritus through overexpression of IL-6 and phosphorylation of Bruton’s tyrosine kinase (BTK) and extracellular signal-regulated kinase (ERK) in the dorsal root ganglion [36]. Since CaP-induced ERK phosphorylation was reduced in IL-6 knockout mice, it is speculated that IL-6 plays an essential role in this mechanism. In addition, the high serum concentration of IL-6 in patients with CKD also suggests that IL-6 plays a critical role in uremic pruritus. However, since IL-6 is involved in most systemic inflammatory responses, IL-6 cannot be a specific factor for CKD-associated pruritus.

Accumulation of P, Ca, Al, and Mg can contribute to pruritus [37,38,39]. Hypercalcemia and hyperphosphatemia were identified as independent risk factors for CKD-associated pruritus [9]. Comparing the serum metabolite profiles of patients with mild and severe CKD-associated pruritus suggested the association between the accumulation of uremic toxins and the onset of pruritus [40]. Various studies showed the altered distribution pattern of accumulated uremic toxin in patients with CKD, which was in line with the fact that the retention of multiple solutes causes uremic syndrome.

### 3.4. Immune System Dysregulation

Immune system dysregulation was suggested as a mechanism of CKD-associated pruritus. This is supported by immunomodulating therapies such as ultraviolet (UV) therapy and tacrolimus to reduce pruritus [41,42]. Kidney transplantation patients on cyclosporine did not suffer from pruritus even when transplant function was lost. 

In addition, several inflammatory markers associated with pruritus increased in patients with CKD-associated pruritus [39,43,44]. The hemodialysis patients with pruritus showed higher levels of serum CRP, IL-6, IFN-γ, and CXCR3 producing CD4 cells [45].

### 3.5. Xerosis

Xerosis is associated with a change in skin pH. Atrophy of the sebaceous glands and thickening of the basement membrane led to dryness of the stratum corneum and increased skin pH. In addition, relief of itching by moisturizing the skin suggests that xerosis is involved in the pathogenesis of CKD-associated pruritus.

### 3.6. Neuropeptide Natriuretic Polypeptide b (NPPB)

Neuropeptide natriuretic polypeptide b (NPPB) is a neuropeptide released by the primary afferent terminal afferent fibers. In 2013, Mishra et al. suggested that NPPB and NPR1, a receptor for NPPB, may play a potential role in itch sensation [46]. NPPB-knockout mice did not respond to pruritogens and intrathecal application of NPPB-induced scratching behavior in the mice. Furthermore, mice in which NPR1-expressing cells were ablated did not respond to histamine-induced itching. 

Initially studied in mice, NPPB/NPR1 signaling was further investigated by Solinski et al. They found that NPPB has similar distribution and function in mice and humans, implying that human itch transmission shares the same itch-signaling molecule [47]. 

In this context, it has been suggested that acute and chronic pruritus in mice can be suppressed through low-molecular-weight NPR1 inhibitors [47]. NPR1 inhibition is now a novel strategy for patients with renal failure [47].

## 4. Treatment of CKD-Associated Pruritus

Unfortunately, treatment guidelines for CKD-associated pruritus have not yet been established. Treatment methods such as emollients, antihistamines, topical steroids, and ultraviolet therapy are a few of the agents currently being used [37]. These treatments are generally helpful in treating itching from various causes and can be tried first because as they do not have serious side effects.

Antihistamines are a frequently used treatment modality used for CKD-aP. There are two groups of antihistamine: one group antagonizes histamine receptors, and another group inhibits histamine release from mast cells. While histamine receptor antagonists such as diphenhydramine and hydroxyzine were not successful, mast cell stabilizers such as ketotifen significantly alleviated uremic pruritus [48,49]. Topical corticosteroids relieve itching in inflammatory skin conditions. However, the effect is unclear when there is no underlying inflammation, and steroids may have side effects when used for a long time. Dry skin is present in the majority of patients undergoing dialysis. This could be due to atrophy of sweat or sebaceous glands or both. Daily topical treatment using rehydrating emollients may alleviate pruritus due to xerosis.

However, as the pruritus worsens, the treatments are often ineffective. To overcome these limitations, research on new treatment strategies is actively being conducted.

### 4.1. Gabapentin or Pregabalin

Several studies evaluated the effects of gabapentin and pregabalin on CKD-associated pruritus [50,51,52,53,54,55,56,57]. Gabapentin and pregabalin are analogs of the neurotransmitter gamma-aminobutyric acid (GABA). Although unclear, their predominant mechanism of action is thought to be the inhibition of the α 2δ subunit of voltage-sensitive Ca^2+^ channel (VSCC), leading to an increased threshold for neuronal excitation by pruritic stimuli [58]. Gabapentin or pregabalin showed a significant improvement in CKD-associated pruritus in several studies [52,53,54,55,57]. One study that compared the efficacy of gabapentin on pruritus of CKD-associated pruritus found that gabapentin did not show a significant difference compared to ketotifen or pregabalin. The most common side effects of gabapentin or pregabalin use are neurological symptoms such as drowsiness, dizziness, fatigue, and sedation [58]. However, since the long-term effects and adverse events have not been thoroughly studied, further long-term studies are required.

### 4.2. TRPV1 and TRPM8 Agonist

The transient receptor potential (TRP) channels expressed by primary sensory neurons and skin keratinocytes are molecular sensors of mechanical, chemical, and thermal environmental stimuli. In recent years, several classes of TRP, including TRPA1, TRPV1, TRPV3, TRPV4, TPM8, and TPRC4, were demonstrated to be involved in itch sensation [59,60]. These findings have led to the development of drugs that target TRP channels for treating chronic itch. 

Activation of TRPV1 and TRPM8 inhibits neurons with pruriceptors through inhibitory neurons such as Bhlhb5-neurons at the spinal cord level (Figure 2) [61]. Capsaicin(TRPV1 agonist) 0.025% cream showed a better effect in reducing uremic pruritus than a placebo [62,63]. Since activation of TRPV1 can evoke neurogenic inflammation, the most common adverse events were a local burning sensation, stinging, and redness. In contrast to capsaicin, activating the TRPM8 channel inhibits the inflammation field [64], which TRPM8 agonists are free from uncomfortable skin sensations. The selective TRPM8 agonist, cryosim-1, has a more prolonged antipruritic effect than menthol, a non-selective TRPM8 agonist [64]. Cryosim-1 was also effective in various circumstances other than CKD-associated pruritus, including the scalp itch [65,66].

### 4.3. Specific Opioid Agonists and Antagonists

The effectiveness of opioid agonists and antagonists are shown in Table 2. Difelikefalin, a KOR agonist, is a treatment for CKD-associated pruritus for hemodialysis patients and was approved by the U.S. FDA in 2021. A double-blind, randomized controlled phase 3 clinical trial recruited 378 patients from 56 institutions in the USA [67]. Patients were randomized into a study group, receiving 0.5 μg/kg intravenous injection of Difelikefalin three times a week for 12 weeks, or a control group receiving a placebo for 12 weeks. It was found that 51.9% of the patients in the study group improved by 3 points or more on the Worst Itching Intensity Numerical Rating Scale (WI-NRS), whereas 30.9% improved in the placebo group (*p* < 0.001). In addition, 37.1% in the study group and 17.9% in the placebo group showed WI-NRS improvement of 4 or more points (*p* < 0.001). Difelikefalin significantly improved the itch-related quality of life after 12 weeks of treatment in the 5-D itch scale and the skindex-10. Adverse events such as diarrhea, dizziness, and nausea were more frequent in the study group than in the control group [67]. 

Nalbuphine, a KOR agonist and MOR antagonist, showed a reduction in the mean visual analog score for pruritus from 4.0 to 1.2 at 180 mg/day and 0.4 at 240 mg/day [68] in an open-label, dose-escalation trial on 15 hemodialysis patients. In an eight-week, randomized, double-blind, placebo-controlled multicenter trial, nalbuphine 120 mg, but not nalbuphine 60 mg, improved pruritus as assessed by a numerical rating scale. This study did not compare nalbuphine with other well-established therapies such as gabapentin or pregabalin. 

Although a few short-term studies suggested that naltrexone, an opioid antagonist, was adequate for uremic pruritus [69], no benefit was demonstrated in a randomized, double-blind, placebo-controlled crossover study [70]. 

Nalfurafine hydrochloride, a KOR agonist, significantly reduced the CKD-associated pruritus [71]. Administration of nalfurafine showed a reduction of pruritus in hemodialysis patients in the phase 3 clinical trial and an open-label long-term study in Japan [71].

### 4.4. Cannabinoids

Cannabinoids consists of a broad range of endogenous and exogenous arachidonic acid derivatives that act on the cannabinoid receptors CB1 and CB2 [72]. In brief, the antipruritic effects of cannabinoids are proposed to be due to a combination of an impact on neuronal activation by stimulation of the CB1 in the central nervous system, transmission through afferent pathways by impacting TRPV1 channels, modulation of immune cells through CB2, and local regulation of keratinocytes and mast cells [72]. According to a study with 21 hemodialysis patients, 81% of patients treated with topical formulations containing two endocannabinoids showed improvement in pruritus after three weeks [73].

### 4.5. Dialysis Modification

A study that evaluated pruritus using the dialysis methods showed that high-flux hemodialysis had a better effect in improving the pruritus compared to low-flux hemodialysis and hemodialysis filtration [74,75]. In addition, combined hemodiafiltration and hemoperfusion were proven more effective than combined hemodialysis and hemoperfusion [76]. This effectiveness is thought to be due to an increase in the removal of β2-microglobulin, inflammatory cytokines, PTH, and methyl guanidine. Another study showed high-permeability hemodialysis to be better than conventional hemodialysis [77].

### 4.6. Others

Phototherapy is widely used to treat pruritus caused by various disorders, for example, atopic dermatitis and psoriasis. A number of studies [78,79,80] have demonstrated the effectiveness of phototherapy on uremic pruritus. In the late 1970s, it was reported that 80–90% of CKD-aP patients treated with Ultraviolet B (UVB) phototherapy showed favorable response in pruritus intensity [78]. A meta-analysis conducted in 1990s suggested that UVB radiation was effective in alleviating uremic pruritus, whereas Ultraviolet A(UVA) phototherapy did not appear to be effective [81]. Surprisingly, Narrowband(NB)-UVB phototherapy did not show a significant effect in reducing pruritus intensity for refractory uremic pruritus compared to a control group [80]. 

Montelukast, a leukotriene receptor antagonist, showed significantly improved CKD-associated pruritus compared to the placebo [82]. In phase 2, a randomized, double-blind clinical trial, nemolizumab, a subcutaneously administered humanized monoclonal antibody against interleukin-31 receptor A, could not meet the primary efficacy endpoint and showed no major safety concerns [83]. In addition, sodium thiosulfate has been reported to improve pruritus in patients without significant side effects [84].

## 5. Clinical Feature of Cholestasis-Associated Pruritus

Bile is a digestive fluid produced by the liver and secreted into the duodenum through the bile duct. Hepatic diseases or extrahepatic biliary duct obstruction can induce abnormal bile secretion and flow, resulting in cholestasis. Cholestasis is accompanied by jaundice, dark urine, and itching. The incidence of cholestatic pruritus varies according to liver disease (Table 3). The pruritus is prerequisite for diagnosis of intrahepatic cholestasis of pregnancy (ICP). Pruritus occurred in 70–80% of cases of primary biliary cholangitis (PBC) 10 years after diagnosis. In addition, the patients of primary sclerosing cholangitis (PSC) have pruritus in 20–40% at presentation and tend to increase with disease course.

Cholestatic pruritus can be generalized or localized, particularly to the palms and soles. It can be exacerbated at night or by psychological stress. The pruritus can hamper the patient’s quality of life, resulting in sleep disturbance or emotional disturbances [71]. The severity does not correlate with the severity of the underlying liver disease, and it can wax and wane. Pruritus can lessen with time in patients with primary biliary cholangitis.

ICP presents in the second or third trimester with the sudden onset of severe pruritus. The pruritus starts on the palms and soles and then delocalizes. It is a hormonally triggered cholestasis in women with a defect in bile acid excretion. Pruritus in intrahepatic cholestasis of pregnancy typically resolves within a few days of delivery [72]. 

A presumptive diagnosis can be made in patients with cholestasis that complain about itching. Although extensive evaluation is generally not required, patients should have a physical examination to rule out the presence of other dermatologic disorders with pruritus. In addition, uremic pruritus should be considered in patients with chronic kidney disease. If the cause of cholestasis is unknown, the patient should undergo additional evaluation with laboratory tests and imaging studies such as ultrasonography.

## 6. Pathogenesis of Cholestasis-Associated Pruritus

Pruritogens of cholestatic pruritus include bile components and various endogenous peptides.

### 6.1. Bile Acid

Bile acid is a steroid synthesized from cholesterol within hepatocytes or the gut. The primary bile acids, such as cholic acid (CA) and chenodeoxycholic acid (CDCA), are synthesized in the liver, and the secondary bile acids, such as deoxycholic acid (DCA), are synthesized in the gut by its microbiome. CA, DCA, and CDCA are known to induce pruritus when injected into healthy humans [91]. CDCA plays a significant role in pruritus induction in biliary duct obstruction. 

Bile acid binding to Takeda G protein-coupled receptor 5 (TGR5), the bile acid receptor, is believed to be important in cholestatic pruritus. In mice, bile acid stimulates the secretion of neuropeptides in the spinal cord, which transmit pruritus by activating TGR5. In addition, bile acids bound to TGR5 activate the TRPA1 to send a signal to the sensory nerve. Therefore, TGR5 is thought to be responsible for cholestatic pruritus in mice [92]. However, a clinical trial found that the TGR5-specific agonist did not induce itch in humans. This led to the discovery of a novel human bile acid receptor, Mas-Related G Protein-Coupled Receptor member X4 (MRGPRX4).

### 6.2. Bilirubin and MRGPRX4 Receptor

Increased levels of bilirubin has been observed in cholestasis, and therefore bilirubin has been suggested as a direct pruritogen. However, serum concentrations of bilirubin is not correlated with the severity of pruritus [93]. For example, in case of anicteric cholestasis, the serum levels of bilirubin is within the normal range, but it is accompanied by severe pruritus [94]. In addition, pruritus is not prominent in the case of Dubin–Johnson syndrome, characterized by elevated serum conjugated bilirubin levels due to mutations in the bilirubin transporter ABCC2 [95]. Similarly, only a tiny percentage of patients with type 1 Crigler-Najjar syndrome, characterized by severely elevated serum unconjugated bilirubin level caused by mutations in the bilirubin–glucuronosyltransferase UGT1A1, had cholestatic pruritus. 

A study suggested that bilirubin can induce pruritus in mice by binding to the Mas-Related G Protein-Coupled Receptor member A1 (Mrgpra1). Furthermore, the human MRGRPX4 receptor expressed in HEK293 cells, which is closer to the Mrgpra1 receptor in mice, was also activated by bilirubin. Also, pruritus was induced when the mouse was injected with the serum of a patient with hyperbilirubinemia. Since there is not enough evidence to support that bilirubin causes pruritus in cholestasis through MRGPRX4 receptor, further studies on the roles of bilirubin and MRGPRX4 in cholestatic pruritus are needed [92].

### 6.3. Substance P (SP)

SP is a peptide composed of 11 amino acids and a substance involved in inflammation, pain, and itching. It is derived from a specific precursor protein called preprotachykinin A, similar to endogenous opioids. SP is evenly distributed in the central and peripheral nervous systems [96]. Serum analysis of liver cirrhosis patients has confirmed that the liver cirrhosis patients have increased levels of SP. Furthermore, the serum of cholestatic mice has elevated levels of SP [97,98]. SP was previously known to act only on the neurokinin receptor NK-1R, but it has recently been found that SP also activates several MRGPRs [99,100]. The itch stimulus is transmitted through itch sensory nerve fibers to the spine and brain. Several studies have reported that chronic pruritus is associated with the substance P/NK-1R [101].

### 6.4. Lysophosphatidic Acid (LPA) and Autotaxin

LPA is a minor, ubiquitous phospholipid that plays essential roles in cell survival, apoptosis, motility, morphogenesis, cell differentiation, and so on [102,103,104]. LPA has been identified as a pruritogen in the serum of cholestasis patients with pruritus, and the activity of the LPA-generating enzyme in these patients was positively correlated with the intensity of itching [105]. Interestingly, intradermal injection of LPA into mice induced scratching. Autotaxin, a serum enzyme that converts lysophosphatidylcholine (LPC) to LPA, is significantly increased in patients with gestational cholestasis and cholestatic pruritus. As the activity of autotaxin showed a positive correlation with the intensity of pruritus, LPA and autotaxin proved to be potent endogenous substances inducing cholestatic itch [105]. LPA was found to promote the activity of LPA5 receptors, phospholipase D, TRPV1, and TRPA1, and the exact molecular mechanism has been elucidated.

### 6.5. Lysophosphatidylcholine (LPC) and TRPV4

A study suggested that LPC, LPA with choline attached to the phosphoric acid moiety, can cause cholestatic pruritus [60]. It was found that LPC is increased in cholestasis patients and mice, activates TRPV4 in keratinocytes, thus activating the sensory nerve that transmits pruritus. Therefore, LPC was recently suggested as a potential candidate for cholestatic pruritus.

### 6.6. Histamine

Histamine, secreted by mast cell degranulation, is the most famous pruritogen, acting by binding to the histamine receptor, H1R or H4R [106]. It was shown that patients with cholestatic pruritus have higher levels of serum histamine [107]. It was suggested that the hyperplasia of mast cells in the liver causes continuous degranulation of mast cells [108]. Bile acids such as CDCA and DCA activate mast cells, leading to histamine degranulation. However, the antihistamine treatment is ineffective in cholestatic pruritus and the lesions of patients with cholestatic pruritus do not have the characteristic rashes caused by histamine, and therefore, histamine is unlikely to be a major pruritogen in cholestatic pruritus.

### 6.7. Bovine Adrenal Medulla 8-22 (BAM8-22)

BAM8-22 is an endogenous peptide consisting of 15 amino acids that activate human Mas-Related G Protein-Coupled Receptor member X1 (MRGPRX1) and mouse Mas-Related G Protein-Coupled Receptor member c11 (Mrgprc11) or Mas-Related G Protein-Coupled Receptor member X1 (MRGPRX1). It induces pruritus through a G protein-α q and 11 subunits (Gαq/11)-dependent pathway [109]. BAM8-22 is one of the by-products of proenkephalin (PENK) that is increased in cholestasis [110]. It was suggested that pruritus is caused by increased levels of BAM8-22 in cholestasis. Indeed, the rise in BAM8-22 and MRGPRX1 in sensory nerves was observed in the mouse model.

### 6.8. Others

Serotonin is a neurotransmitter related to physical and mental comfort and a pruritogen [111]. The serotonin system was thought to mediate cholestatic pruritus, as evidenced by the use of serotonin type 3 receptor antagonists and selective serotonin reuptake inhibitors [112]. Pruritus was induced two weeks after serotonin was injected intra-dermally into the back of the neck and cheeks of mice that underwent bile duct blockage, whereas injection of saline did not induce pruritus [113]. Further studies on the role of serotonin in cholestatic pruritus are needed.

According to a study, the expression of inducible NO synthase (iNOS) was increased in a mouse model of cholestasis. Meanwhile, pruritus in these mice was suppressed by the NO synthase inhibitor [114].

## 7. Treatment of Cholestasis-Associated Pruritus

### 7.1. Treatment for Underlying Disease

**Figure 3:**
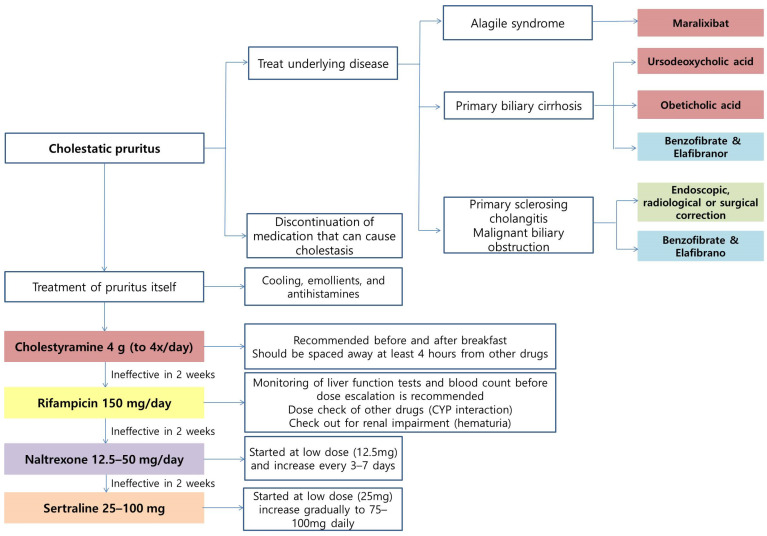
The treatment algorithm of cholestatic pruritus. Green: Treatment of bile duct obstruction; Red: inhibit bile acid synthesis or increase excretion (prevent reabsorption); Purple: opioid antagonist; Blue: improving overall liver function; Orange: regulation of neurotransmitters; Yellow: excretion and detoxification of pruritogens.

The first step in treating patients with cholestatic pruritus is treating the underlying disease and bile duct obstruction (Figure 3). Endoscopic, radiological, or surgical correction can be applied in primary sclerosing cholangitis (PSC) and malignant biliary obstruction. Discontinuation of medication that can cause cholestasis should be attempted first. In addition, general measures such as cooling, emollients, and antihistamines can be helpful for pruritus, though antihistamines are mostly ineffective.

### 7.2. Ursodeoxycholic Acid (UDCA) and Obeticholic Acid (OCA)

The naturally occurring human bile acids are mostly hydrophobic except for a few such as UDCA, which is a hydrophilic bile acid. It can help expand the hydrophilic component of the bile pool, thereby decreasing the overall bile acids and increasing the secretion of bile acid. 

In the case of primary biliary cirrhosis (PBC), UDCA is the treatment of choice in a dose of 10–15 mg/kg/daily, which delays progression to liver cirrhosis and enhances survival [115,116]. UDCA competes for intestinal absorption and increases hepatic clearance of bile acids. Approximately 40% of patients did not respond to UDCA [117]. OCA is a bile acid analog, a selective Farnesoid X receptor (FXR) agonist. FXR represses bile acid uptake, suppresses bile acid synthesis, and stimulates biliary secretion. However, OCA administration was also reported to exacerbate pruritus in a dose-dependent manner [118].

### 7.3. Benzofibrate/Elafibranor

A clinical trial of benzofibrate, a peroxisome proliferator-activated receptor (PPAR) agonist, was conducted in patients with PBC and PSC. Pruritus in the benzofibrate-treated group showed more improvement compared to the placebo group [119]. In addition, benzofibrate can improve liver fibrosis and alleviate fatigue [120]. Elafibranor, another PPAR α/δ agonist, also underwent a phase 2 clinical trial, but the effect on pruritus was found effective only in the second half of treatment. These clinical trials showed that PPAR agonists could be utilized as new therapeutic drugs for treating cholestatic pruritus by improving overall liver function.

### 7.4. Cholestyramine

Cholestyramine is a bile acid sequestrant that binds bile in the GI tract to prevent its reabsorption. It removes pruritogens from the enterohepatic cycle by preventing the reuptake. 

The required dose is 4 g four times per day. Resins should be spaced at least 4 h from other drugs to prevent binding and drug interactions, and the morning dose is preferred [115,121]. Poor tolerance is often an issue due to the bad taste and complaints such as constipation and bloating [115].

### 7.5. Rifampicin

Rifampicin, a pregnane X-receptor (PXR) agonist, can be prescribed if the cholestyramine is ineffective. It stimulates the excretion of pruritogens and detoxification and alters intestinal metabolism with an antimicrobial effect. Rifampicin was safe and effective, relieving pruritus in up to 77% of the patients as compared to placebo or alternatives in the meta-analyses [122]. It was shown that 150 mg rifampicin monitoring liver function tests and blood count before dose escalation is recommended [123] because drug-induced hepatitis and significant liver dysfunction were reported [124,125]. Other reported adverse effects include disruption of vitamin K metabolism, hemolytic anemia, thrombocytopenia, renal impairment, and drug interactions.

### 7.6. Naltrexone

As discussed, when MOR is suppressed, itching is reduced. Naltrexone, the opioid antagonist, is an alternative treatment when rifampicin does not have an effect within two weeks. It should be started at a low dose (12.5 mg) and increased every 3–7 days. When naloxone is administered intravenously, it quickly removes the opioids from the receptors in the brain, and therefore it can cause a withdrawal-like syndrome. Thus, oral therapy should be preceded. Naltrexone hepatotoxicity was reported but is uncommon [116].

### 7.7. Selective serotonin Reuptake Inhibitor (SSRI)

Sertraline can be considered an alternative treatment for resistant patients. The starting dose is 25 mg per day, increasing gradually to 75–100 mg per day. It can reduce pruritus by altering neurotransmitter concentrations within the central nervous system. Adverse events include dry mouth, nausea, dizziness, and diarrhea [126].

### 7.8. Maralixibat 

Maralixibat is the first treatment approved for cholestatic pruritus in patients with Alagille syndrome and was approved by the US FDA in September 2021. Alagile syndrome is a rare genetic disorder in which bile builds up in the liver due to paucity of interlobular bile ducts. Alagille syndrome causes severe cholestasis, resulting in liver damage as bile accumulates in the liver. Maralixibat inhibits the enterohepatic circulation of bile acids by inhibiting the reabsorption process via the apical sodium-dependent bile acid transporter (ASBT). It can increase the excretion of bile acids and relieve pruritus by lowering serum bile acids and cholesterol levels [127]. In a phase 2 clinical trial, a significant improvement in pruritus was achieved after three weeks of treatment [127]. However, follow-up studies are needed to determine its effect on other disorders with cholestatic pruritus. Nevertheless, Maralixibat is the only FDA-approved treatment to this date that can selectively suppress cholestatic pruritus.

## 8. Conclusions

As pruritus caused by systemic diseases impairs patients’ quality of life and is difficult to treat, the current knowledge is drawing more attention. Unknown mechanisms are being studied through animal or human studies today, and various treatments are being tested through clinical trials. This review explains the specific systemic diseases that cause pruritus and summarizes the current knowledge. In the future, further research will help understand the mechanism and develop new strategies.

## Figures and Tables

**Table 1:** Risk factors of patients with CKD associated pruritus.

Risk Factors	Characteristics
**Inadequate dialysis**	High level of BUN was a significant risk factor for severe uremic pruritus [9,10,11].
**Hyperparathyroidism product**	Hyperparathyroidism, the product due to the alteration of metabolism, can be involved in the pathogenesis and risk factor [12,13].
**Elevated calcium x phosphorus product**	Calcium and phosphate, the product due to the alteration of metabolism, can be involved in the pathogenesis and risk factor [9,14,15].
**Xerosis**	Xerosis is a common complication in HD patients and has an impact on the severity of itch [2,16,17].
**Elevated serum magnesium and aluminum concentrations**	Magnesium and aluminum, the product due to the alteration of metabolism, can be involved in the pathogenesis and risk factor [18,19,20].

**Table 2:** Effectiveness of opioid agonists and antagonists.

Drug	Class	Effectiveness
Difelikefalin	KOR agonist	Worst Itching Intensity Numerical Rating Scale (WI-NRS) improved by 3 points or more in 51.9% of the patients in a double-blinded, randomized, controlled phase 3 clinical trial Itch-related quality of life in the 5-D itch scale and the skindex-10 significantly improved in the trial
Nalbuphine	KOR agonist MOR antagonist	Mean visual analog scale for pruritus significantly improved in an open-label trial (4.0 to 1.2 at 180 mg/day and 0.4 at 240 mg/day) Numerical rating scale for pruritus improved with nalbuphine 120mg, but not nalbuphine 60mg in a randomized, double-blind, placebo-controlled multi-center trial
Naltrexone	Nonselective opioid antagonist	Beneficial effect not demonstrated in a randomized, double-blind, placebo-controlled crossover study
Nalfurafine hydrochloride	KOR agonist	Significant reduction of pruritus shown in phase 3 clinical trial and open-label long-term study

**Table 3:** Frequency of cholestatic pruritus according to the type of liver disease.

Specific Liver Disease	Characteristics	Incidence
**Intrahepatic cholestasis of pregnancy**	Characterized by pruritus and an elevation in serum bile acid levels, developing in the second or third trimester and resolving after delivery.	100% [85]
**Primary biliary cholangitis**	It is common in females between the ages of 30 and 65 years. Pruritus often precedes the development of jaundice and can be accompanied by skin findings.	70–80% by 10 years [86,87]
**Primary sclerosing cholangitis**	Chronic progressive disorder characterized by inflammation and fibrosis. The incidence increases as the disease progresses.	20–40% (Initial) [88,89]
**Malignant biliary tract obstruction**	It can be due to the presence of tumor in the gallbladder, bile duct, ampulla, duodenum, or pancreas.	45% [90]
**Chronic viral** **hepatitis**	The hepatitis B and C viruses can cause chronic hepatitis and can lead to cirrhosis, liver failure, and liver cancer with pruritus.	20% [90]
**Nonmalignant biliary tract obstruction**	Choledocholithiasis, cholecystitis, and stricture of commone bile duct can cause pruritus.	17% [90]
**Cirrhosis**	Patients with decompensated cirrhosis can present with jaundice, pruritus and gastrointestinal bleeding.	7% [90]

## Data Availability

Not applicable.

## Abbreviations

CA	Cholic acid
CaP	Calcium phosphate
CDCA	Chenodeoxycholic acid
CKD	Chronic kidney disease
DCA	Deoxycholic acid
DOR	δ-opioid receptor
GABA	Gamma-aminobutyric acid
KOR	κ-opioid receptor
LPA	Lysophosphatidic acid
LPC	Lysophosphatidylcholilne
MOR	μ-opioid receptor
MRGPRX4	Mas-Related G Protein-coupled Receptor member X4
SP	Substance P
TGR5	Takeda G protein-coupled receptor 5
TRP	Transient receptor potential
UV	Ultraviolet
